# A Rare Case of Colorectal Cancer With Delayed Metastasis to the Duodenum

**DOI:** 10.1155/crgm/6679555

**Published:** 2025-01-13

**Authors:** Ammad Javaid Chaudhary, Abdulmalik Saleem, Muhammad Shahzil, Nosheen Hafeez, Taher Jamali, Brian Ginnebaugh

**Affiliations:** ^1^Department of Internal Medicine, Henry Ford Hospital, Detroit, Michigan 48202, USA; ^2^Department of Internal Medicine, Penn State Health Milton S Hershey Medical Center, Hershey, Pennsylvania 17033, USA; ^3^Department of Internal Medicine, Baptist Health-UAMS, Little Rock, Arkansas 72205, USA; ^4^Department of Gastroenterology and Hepatology, Henry Ford Hospital, Detroit, Michigan 48202, USA

## Abstract

Colorectal cancer (CRC) continues to be a significant global health issue contributing to a high mortality rate. Despite advancements in treatment, the risk of recurrence remains due to inherent mutations and the rapid turnover of intestinal mucosa. We present an exceptionally rare case of CRC metastasis to the duodenum in a 42-year-old female who has been compliant with postsurgical surveillance. Despite previous negative surveillance results, elevated CEA levels and a 3-cm mesenteric mass were detected, raising concerns for carcinoma, which was later confirmed by biopsy. The tumor board deemed her ineligible for surgery due to vascular involvement, leading to palliative care and an attempt at neoadjuvant therapy. Vigilant monitoring is crucial for early detection and intervention.

## 1. Introduction

Colorectal cancer (CRC) continues to be the second-most common cause of cancer-related deaths worldwide, causing approximately 850,000 deaths annually [1]. Furthermore, the incidence of CRC is rising among the young [2]. Approximately 50% of the patients diagnosed with nonmetastatic CRC develop metastasis through their disease. Therefore, it is crucial to identify recurrence and metastasis early to decrease mortality [3]. The liver is the most common site of CRC metastasis, followed by the lung, regional lymph nodes, peritoneum, and rarely the duodenum [3–5].

Recent advancements have yielded new treatment options for primary and metastatic CRC including surgical resection, radiotherapy, and chemotherapy [6]. Targeted monoclonal antibodies and immune checkpoint inhibitors have proven to improve survival rates [7]. Regardless of the treatment of CRC, there is a tremendously high risk of recurrence of colorectal carcinoma due to inherent mutations and high turnover rate of the intestinal mucosa. As a result, regular posttreatment surveillance to identify recurrences at a curable stage is imperative [8].

We present a rare case of CRC metastasis to the duodenum in a patient who is compliant with its postsurgical surveillance.

## 2. Case Presentation

A 42-year-old woman presented with a 2-week history of persistent fatigue, shortness of breath, presyncope, and hematochezia. Notably, the patient's medical history included a prior diagnosis of stage IVA ileocecal adenocarcinoma with liver metastasis at the age of 39. She subsequently underwent a robotic right hemicolectomy and partial hepatectomy. Pathology from this initial liver and colon resection demonstrated metastatic moderately differentiated adenocarcinoma and pT3N0M1a invasive moderately differentiated adenocarcinoma in the cecum, respectively. Both the liver and colon margins were negative for dysplasia and adenocarcinoma. Zero out of 16 excised lymph nodes demonstrated metastasis. Subsequent surveillance, which included annual carcinoembryonic antigen (CEA) levels and computed tomographic (CT) scans consistently revealed no evidence of disease recurrence over the next 35 months. The patient was asymptomatic during this time period.

**Figure 1:**
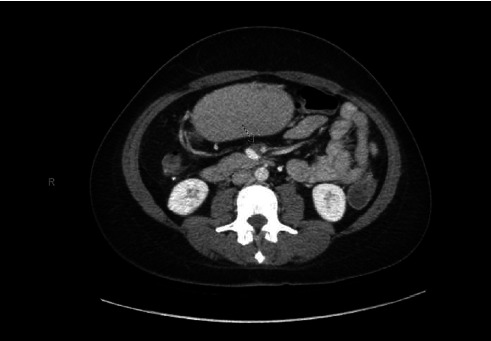
CT scan of the abdomen and pelvis revealing a 3-cm central mesenteric mass invading the duodenum, encasing the superior mesenteric artery (SMA), and abutting the superior mesenteric vein (SMV). A new 8-mm lesion in the peripheral hepatic segment 5/8 is also identified (indicated by the arrow).

**Figure 2:**
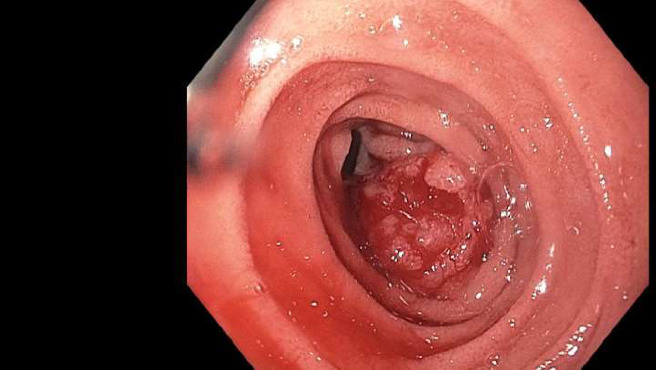
Esophagogastroduodenoscopy (EGD) image showing a 3-cm fungating mass in the second part of the duodenum, which was confirmed to be carcinoma on biopsy.

**Figure 3:**
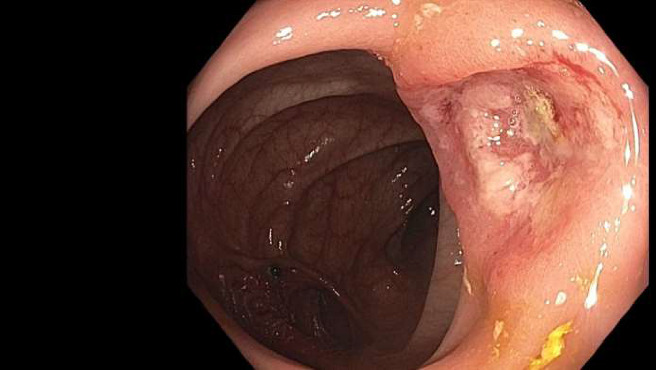
Colonoscopy findings indicating diverticulosis, nonbleeding hemorrhoids, and ulcers in the colon.

However, upon presentation, the patient exhibited a hemoglobin level of 5.9 mg/dL, necessitating transfusion of 2 units of packed red blood cells. Notably, CEA levels were elevated at 11.1 ng/mL, compared to 13.7L and 6.7 ng/nL one and two years prior, respectively. A CT scan of the abdomen and pelvis with intravenous contrast unveiled a 3-cm central mesenteric mass (Figure 1). This mass invaded the duodenum, encased the superior mesenteric artery (SMA), and abutted the superior mesenteric vein (SMV). Furthermore, a new 8-mm lesion in the peripheral hepatic segment 5/8 (Figure 1—red arrow) was also identified. An esophagogastroduodenoscopy (EGD) showed a 3-cm fungating mass in the second part of the duodenum concerning for carcinoma, which was later confirmed on biopsy (Figure 2). To note, the patient did not have a previous endoscopy on record. The colonoscopy identified diverticulosis, nonbleeding hemorrhoids, and ulcers in the colon (Figure 3). In addition, the patient was found to have mutant KRAS.

The case was presented to the tumor board, which collectively determined that the patient was not a suitable candidate for surgical resection due to vascular involvement. The patient was subsequently started on neoadjuvant FOLFOX and is currently undergoing a total of six cycles, which she is tolerating well.

## 3. Discussion

Among patients with CRC, the most common cause of death is recurrence and metastatic spread of the disease. Twenty-five percent of the patients diagnosed with CRC have widespread metastatic disease at the time of diagnosis (synchronous), and 50% of the patients with nonmetastatic disease on diagnosis end up developing metastasis (metachronous), as seen in our patient. The hypotheses used to explain metastatic spread are the “seed-and-soil” and “anatomical/mechanical” spread hypotheses, which explain spread to the liver, lung, regional lymph nodes, and peritoneum. It also depends on the type of cancer, with well-differentiated adenocarcinomas metastasizing more often [9].

Metastasis from colorectal carcinoma to the duodenum is exceedingly rare and sparsely documented [10–12]. By contrast, peritoneal metastasis is relatively common in CRC, occurring in 8%–20% of cases. A population-based study reports the incidence of peritoneal carcinomatosis as 8.3% (4.3% synchronous and 4.2% metachronous). Another study notes that 20% of metastatic CRC cases present with synchronous peritoneal metastases [13, 14]. Rates of intra-abdominal patients younger than 60 years and survive five or more years after the diagnosis of colon cancer have a two-fold increased risk of small intestinal cancer, according to the SEER programs report [15]. As with metastasis, there are also two types of recurrence in colorectal carcinoma: Type 1, where the micrometastatic cancer cells proliferate after destabilization from their dormant stage, and Type 2, when there is the growth of the residual unresected tumor cells and their spread to the distant sites [16]. Thirty percent to forty percent of the patients develop a recurrence of the disease in the first 2 years postsurgical resection of the tumor [17].

Surgical resection with concomitant chemotherapy/biological agents is the treatment of choice for patients with local, locoregional, and metastatic disease [18]. However, despite the newer treatment options, only 20% of the patients achieve remission, with 60%–70% developing local or distant metastasis [19]. Considering that there is a high probability of recurrence during the first 5 years following the treatment, there is a need for guidelines to screen for tumor recurrence to avoid complications, missed lesions or, rapidly growing cancers, as observed in our young female patient [20].

The standard surveillance method for CRC after resection is serial CEA measurement and routine colonoscopy. The American Society of Clinical Oncology recommends yearly CT scans for 3 years, along with CEA and colonoscopy, for the surveillance of CRC. MRI is widely used for TNM staging and restaging postchemotherapy; however, its significance in detecting recurrence is not established yet [21]. A prospective RCT showed increased cost and no benefit over 6-monthly 18F-FDG PET/CT scan at 3 years follow-ups of the patients [22]. Therefore, there is consensus on not using PET scans for screening for recurrence of CRC unless there are consistent elevations in CEA after the therapy or to confirm the absence of metachronous disease after surgical resection. However, a newer meta-analysis shows some benefit of PET scans in detecting relapse of CRC even in patients with normal CEA levels [23]. During a prospective study, it was found that the sensitivity of PET/CT in detecting the recurrence of CRC was 94.6%, and the specificity was 83.3% [24]. Furthermore, patients with KRAS mutations are at a higher risk of CRC recurrence [25].

Recurrence occurred in our patient despite appropriately scheduled surveillance, highlighting that it is imperative that clinicians remain on high alert when assessing these patients. The recent literature favors more frequent surveillance in patients with a RAS mutation in the setting of surveillance of colorectal liver metastasis resection [21]. Further studies are required to comment on tailoring surveillance intervals in the unique setting of duodenal metastasis.

## Data Availability

All data relevant to the findings of this study are included within the manuscript. No additional data were withheld, ensuring complete transparency and reproducibility of the results presented. For any further inquiries or access to specific data points, readers are encouraged to contact the corresponding author.
